# Relationship between the Characteristics of Bread Wheat Grains, Storage Time and Germination

**DOI:** 10.3390/plants11010035

**Published:** 2021-12-23

**Authors:** Dmitry A. Afonnikov, Evgenii G. Komyshev, Vadim M. Efimov, Mikhail A. Genaev, Vasily S. Koval, Peter U. Gierke, Andreas Börner

**Affiliations:** 1 Institute of Cytology and Genetics, Siberian Branch of the Russian Academy of Sciences, 630090 Novosibirsk, Russia; delkom07@gmail.com (E.G.K.); efimov@bionet.nsc.ru (V.M.E.); mag@bionet.nsc.ru (M.A.G.); kovalvs@icg.sbras.ru (V.S.K.); 2 Faculty of Natural Sciences, Novosibirsk State University, 630090 Novosibirsk, Russia; 3 Kurchatov Genomics Center, Institute of Cytology and Genetics, Siberian Branch of the Russian Academy of Sciences, 630090 Novosibirsk, Russia; 4 Landesanstalt für Landwirtschaft und Gartenbau, Dezernat 42, Prüf- und Anerkennungsstelle für Saat- und Pflanzgut, Schiepziger Str. 29, 06120 Halle (Saale), Germany; peterulrich.gierke@llg.mule.sachsen-anhalt.de; 5 Leibniz Institute of Plant Genetics and Crop Plant Research, Corrensstr. 3, OT Gatersleben, 06466 Seeland, Germany; boerner@ipk-gatersleben.de

**Keywords:** wheat grains, seed aging, image analysis, seed traits, seed coat color, germination

## Abstract

Seed storage is important to farmers, breeders and for germplasm preservation. During storage, seeds accumulate damage at the structural and metabolic level, which disrupt their function and reduce resistance to adverse external conditions. In this regard, issues related to seed aging prove to be relevant for maintaining the viability of genetic collections. We analyzed morphological characteristics of grains and their coat color for 44 recombinant inbred lines (RILs) of bread wheat grown in four different seasons, 2003, 2004, 2009 and 2014. Our investigations were performed in 2020. For 19 RILs from the same seasons germination was evaluated. Our results demonstrate that genotype significantly affects the variability of all seed traits, and the year of harvesting affects about 80% of them (including all the traits of shape and size). To identify the trend between changes in grain characteristics and harvesting year, we estimated correlation coefficients between them. No significant trend was detected for the grain shape/size traits, while 90% of the color traits demonstrated such a trend. The most significant negative correlations were found between the harvesting year and the traits of grain redness: the greater the storage time, the more intensive is red color component for the grains. At the same time, it was shown that grains of longer storage time (earlier harvesting year) have lighter coat. Analysis of linear correlations between germination of wheat seeds of different genotypes and harvesting years and their seed traits revealed a negative linear relationship between the red component of coat color and germination: the redder the grains, the lower their germination rate. The results obtained demonstrate manifestations of metabolic changes in the coat of grains associated with storage time and their relationship with a decrease of seed viability.

## 1. Introduction

Seed storage is important to farmers, breeders, and industries interested in seed processing and commercial trade but also for germplasm preservation. During storage, seeds accumulate damage at the structural and metabolic levels, which disrupt their function and reduce resistance to adverse external conditions [1,2]. External conditions affects these processes: higher temperatures and humidity have a negative effect on the seed viability and accelerate their deterioration [3]. However, even when stored under special genebank conditions, seeds gradually lose their ability to germinate [4,5,6]. In this regard, issues related to seed aging prove to be relevant for maintaining the viability of genetic collections.

The processes associated with grain aging are studied at the biochemical level [7,8,9], which allows to determine their molecular mechanisms and, if possible, reduce the impact of damaging factors during storage [10]. There is also active research to find genes associated with seed longevity [11,12,13]. This allows to identify the genes that contribute to a longer storage of grains and the mechanisms of longevity control at the genetic level.

One of the ways to study physiological properties of grains under the influence of various factors is to assess their morphology and the color, for which methods of digital image analysis are actively used [14,15,16,17,18,19]. These methods do not require special expensive equipment, at the same time they allow to assess the degree of change in the parameters of grains quantitatively and to identify among them the ones most closely related to their physiological state, including aging [20,21]. Digital image analysis was used to identify changes of the seed coat color, described as browning, under controlled deterioration in lentil (*Lens culinaris* Medick.) suggesting that the progressive and continuous deterioration process can be monitored with color change [22]. The seed coat browning effect was likewise detected for cucumber (*Cucumis sativus* L.) after ten days storage under decreased moisture content and elevated temperature conditions [20]. Color changes were also detected after accelerated aging for *Medicago sativa* and *Onobrychis viciifolia* seeds [21].

Most work on the study on longevity of grains are based on artificial short-term exposure to factors such as high temperature and humidity [23]. This allows for fast activation of the metabolic processes in the grains, similar to the aging [20,22]. The study of seed aging under natural conditions, allows to investigate the processes that occur under real conditions of maintenance as e.g., in genebanks, but these studies are difficult due to the need for long-term maintenance of grains in storage. To the best of our knowledge, no systematic investigation of quantitative seed characteristics such as size/shape/color has been performed yet for seeds stored for several years in genebank conditions.

In this paper, we analyze the morphological characteristics of grains and their coat color for 44 bread wheat recombinant inbred lines (RILs) of the International Triticeae Mapping Initiative (ITMI) mapping population. Plants were grown in four different seasons, 2003, 2004, 2009 and 2014. Our studies were performed in 2020. For 19 samples grown in the same seasons, germination was evaluated. Using the method of image analysis, we evaluated seed morphometric and color characteristics. We showed that color but not size/shape characteristics, have a significant linear relationship with storage time and seed germination.

## 2. Results

Below, we will describe the results of our analysis of seed size/shape/color characteristics for 44 RILs harvested in different seasons. We will evaluate the similarity of various traits between each other, perform cluster analysis of RILs in the space of these traits, evaluate the influence of harvesting year and the genotype on the trait values. Finally, we will estimate correlation between wheat seed traits, the harvesting year and the germination rate to determine what traits are associated with deterioration due to seed storage and viability.

### 2.1. Seed Traits Cluster Analysis

**Figure 1:**
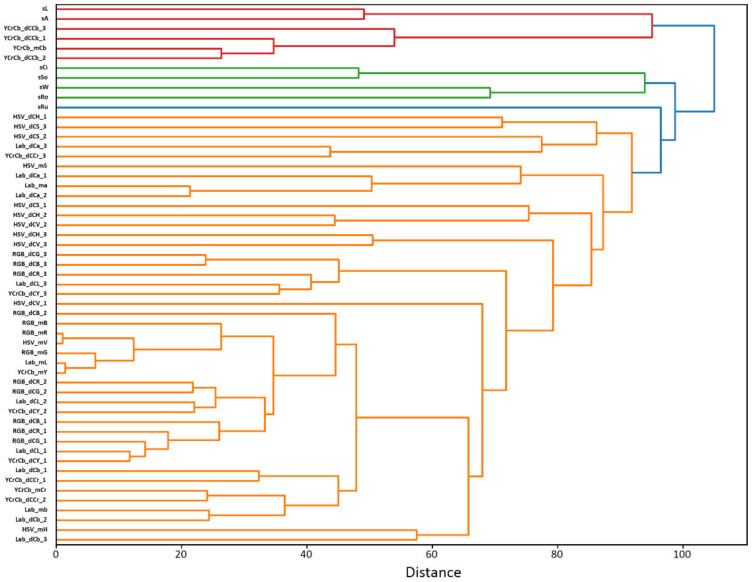
Clustering of wheat grain traits based on the analysis of 44 RILs from ITMI population. Four clusters of traits are highlighted with different colors: large cluster of color traits (orange), seed shape traits (green), traits of seed size and Cb component of the YCrCb color space (red), rugosity (blue).

The results of estimation of the mean values of parameters and correlation coefficients between them in the sample of 44 RILs are given in Supplementary File S1 and Supplementary File S2, respectively. The results of clustering of the 55 grain characteristics are shown in Figure 1.

Several clusters of traits can be distinguished in the diagram of Figure 1. The first, the largest one, shown in orange, includes only the characteristics of the grain color. In this cluster we can distinguish several sub-clusters. First of all, this is a group of features associated with average values of R,G,B components. Close to it are a number of features of other color spaces, reflecting mainly average values of color brightness or luminosity (YCrCb_mY, HSV_mV, Lab_mL). The corresponding components of the first and second dominant colors (RGB_dCB_2, RGB_dCR_2, RGB_dCG_2, Lab_dCL_2, YCrCb_dCY_2, RGB_dCB_1, RGB_dCR_1, RGB_dCG_1, Lab_dCL_1, YCrCb_dCY_1) are located closely to them. Traits of this cluster, therefore, can be attributed to the characteristics of the brightness of grain color.

Next is a group of parameters characterizing the color component b* and Cr for the L*a*b* and the YCrCb spaces, respectively (Lab_mb, Lab_dCb_1, Lab_dCb_2, YCrCb_mCr, YCrCb_dCr_1, YCrCb_dCr_2). These parameters characterize the color change from blue to yellow (L*a*b* component b*), or from green to red (Cr). Next to this cluster are the average values of the HSV hue component (HSV_mH) and Lab_dCb_3.

A large set of parameters that do not form a distinct grouping includes components for the second and third dominant colors (RGB_dCG_3, HSV_dCH_3, etc.). The analysis of mean values of color components of dominant colors (Supplementary File S1) shows that for the color spaces in which one of the components is related to brightness (V for HSV, L for Lab, Y for YCrCb) the three dominant colors of seeds have large differences in these components (dozens of units). For the other two components characterizing color tone these differences are much smaller (up to ten units). Thus, we can conclude that the three dominant colors reflect, to a greater extent, the differences in illumination of various parts of the grain surface rather than the heterogeneity of its color. The unstructured group of features also includes the a* component mean values of the L*a*b* space and the S component of the HSV space (Lab_ma, HSV_mS).

The grouping of the above parameters on the tree agrees well with the Pearson correlation coefficients estimates given in the Supplementary File S2. The correlation coefficients between RGB component values and brightness characteristics YCrCb_mY, HSV_mV, Lab_mL (neighbors in the cluster diagram) exceed 0.8. For parameters Lab_ma, HSV_mH (distant from the R,G,B cluster) the correlation coefficients are negative and significant.

The shape traits (sCi, sSo, sW, sRo) characterizing the roundness/elongation fall into a separate group. The values of rugosity (sRu) is connected to a large cluster with the characteristics of grain color. Apparently, this may be due to ruggedness of the grain boundary (high sRu value) which results in a blurring and contributes to the values of the seed color components.

Of interest is the cluster including the values of the grain length and its area on the image. Both parameters are closely related (Pearson correlation coefficient is 0.81, *p* ≈ 0). However, this cluster also includes values of the color component Cb of the YCrCb scale, both the average, YCrCb_mCb, and values of the three dominant colors (YCrCb_dCb_1, YCrCb_dCb_2, YCrCb_dCb_3). This connection is reflected also by Pearson correlation coefficients: their values between sL and the specified color parameters are 0.37, 0.38, 0.37 and 0.27, respectively, the *p*-values are close to 0 (Supplementary File S2).

The data obtained indicate that the most grain color characteristics form one group of features, while the size/shape characteristics form another. Nevertheless, there are significant statistical relationships between some features of these groups (for example, between the size and the Cb component of the YCrCb space).

### 2.2. Clustering of RILs by Grain Properties

**Figure 2:**
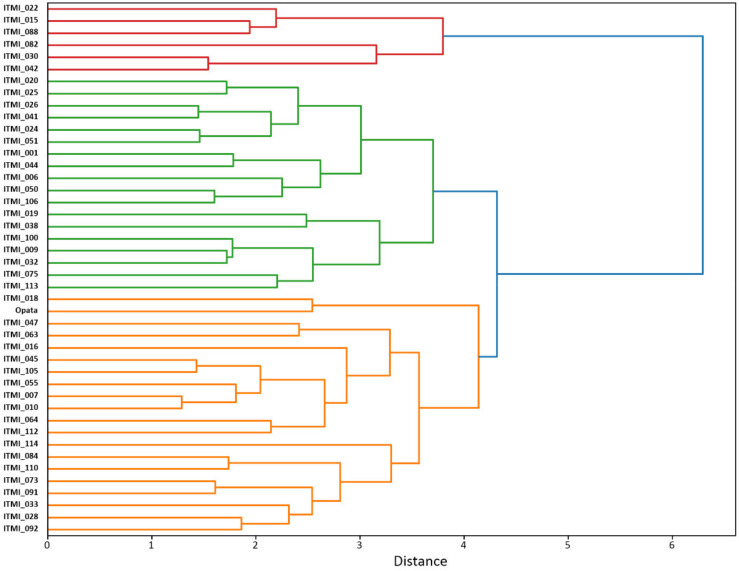
RILs clustering diagram in the space of the grain traits. Three distinct clusters shown by different colors, orange, green and red.

We clustered the genotypes of the ITMI population using their mean values of the grain characteristics. The results are shown in Figure 2.

The diagram shows that the lines have decomposed into three main clusters (marked orange, green, and red in Figure 2). These clusters are clearly visible in the LDA biplot diagram for the color and size or shape traits (Supplementary File S3, Figures S1 and S2).

In first diagram (Supplementary File S3, Figure S1), RILs from the red cluster are located on the right, the green cluster on the left and upper part of the plot, and the accessions from the orange cluster are located between them. These data show that the first axis is associated predominantly with yellow to green color changes: it is positively correlated with such traits as RGB_mG, the b* component of the L*a*b* space (Lab_mb), which determines yellow-green coloration. Traits with negative correlation between this axis are represented by the components associated with the Cb parameter of the YCbCr scale (YCbCr_mCb). The second axis is associated with the component a* of the L*a*b* space (Lab_ma) (negative values) and the Cb parameter of the YCbCr scale (positive values). This component reflects change of seed coat from red (negative values) to yellow (positive values). Based on this, the grains of the red cluster accessions can be characterized by lighter shade with somewhat greener tone, while the green cluster accessions can be characterized by darker yellow shade. Color tone of seeds from orange cluster is intermediate.

The biplot diagram in the plane of two main axes for the grain size and shape features is shown in Supplementary File S3, Figure S2. Two main axes on this plot almost completely coincide with the traits of grain length (Axis 1 negative direction) and width (Axis 2). The direction from the lower left to the upper right corner in this diagram corresponds to an increase in roundness (sRo). Examples of two accessions contrasting in this traits are ITMI_016 (elongated grains) and ITMI_028 (rounded grains), see Figure S3 in the Supplementary File S3.

The direction from the lower right corner to the upper left corresponds to an increase in grain area (size) (Supplementary File S3, Figure S2). It can be seen that the grains of the three clusters are distributed along this direction from a smaller size (red cluster dots), to a larger size (green cluster dots). The orange cluster genotypes are located in between. However, the overlap between these three clusters is remarkable. This implies that variations of the seed shape/size traits are wide.

These data characterize the differences in grain size/shape and coloration traits in different ITMI lines. They reflect the tendency for larger grains to have darker shade and for smaller grains to have lighter shade, which is generally consistent with the clustering of traits and the location of characteristics associated with the Cb component of the YCrCb scale in the cluster of grain size traits (Figure 1).

### 2.3. Analysis of Variance for Grain Traits

To determine the significance of the influence of genotype and grain storage time on the characteristics of grain size/shape and color, analysis of variance was performed. Two factors, genotype and crop year were considered independently, significance was estimated at *p* < 0.05. The results are shown in Supplementary File S4.

The analysis demonstrated that genotype had a significant effect on all of the analyzed traits. For the second factor, harvesting year, the number of such traits was 43 (80%). It should be noted that most traits for which the influence of harvesting year is not significant include the components of the 3rd dominant color for different color spaces. The only non-color seed trait among them is rugosity.

### 2.4. Analysis of Linear Relationships between Harvesting Year and Grain Traits

To determine whether there is a linear correlation between grain traits and harvesting year, we estimated Pearson correlation coefficients between these quantities. We hypothesized that the systematic trend associated with the harvesting year may reflect changes in grain characteristics associated with their storage duration. We used four types of harvesting year coding as described in Section 4, and before analyzing the correlations, we subtracted from the trait values their averages across accessions. The results are given in Supplementary File S5.

First, it is noteworthy that the correlation coefficients between grain traits and various harvesting year encodings are close, the difference is observed mainly in the second decimal digit. Thus, for the trait Lab_ma (the a* component of the L*a*b* space) and coding Year01 the correlation coefficient is −0.69, for coding Year −0.66, for coding YearRank −0.64. This trait has one of the highest in absolute value and negative correlation coefficients. For the trait sL (seed length) all estimates of correlation coefficients with the harvesting year encodings are low in absolute value: for coding Year01 0.00, for coding Year 0.06, for coding YearRank 0.02. This indicates stability of our estimates of correlation coefficients based on three different harvesting year encodings. Note, the absolute values of the correlation coefficient between the YearRank are lowest among others. For 41 out of 55 grain traits, the correlation coefficient for the YearRank was less in absolute value than for any other encodings (for Year01 this number is 8, for Year it is 0). That is why we chose this encoding to test the significance of the Pearson correlation with grain traits by randomization tests.

The results of randomization tests showed that for 45 out of 55 traits, the correlation between YearRank and grain traits is significant for both bootstrap and permutation tests (Supplementary File S6). For example, for the Lab_ma the correlation coefficient in the real sample was −0.64, the minimum values in the permutation/bootstrap experiments were −0.07 and −0.06, respectively, the maximum values were 0.06 and 0.06. Thus, for this trait, the linear relationship with harvesting year is highly significant. For ten traits, the relationship is not significant according this condition. For example, for the length trait sL (real value *r* = 0.02) the *r* minimum values in the permutation/bootstrap experiments were −0.06 and −0.05, respectively, the maximum values were both 0.06.

The data for a number of characteristics whose real correlation coefficients with the YearRank exceed the absolute value of 0.5 (~10 times the threshold from randomization tests) are shown in Table 1. For these characteristics, the ANOVA test showed a significant relationship with the harvesting year as well.

Most of the grain color traits, 44 out of 48 (90%), show a significant relationship with the harvesting year. Among the size/shape traits, only one, rugosity, demonstrates a significant relationship with the year of harvest. For sRu, the correlation coefficient with YearRank in the real sample was 0.08, the maximum and minimum values for permutations were −0.05 and 0.06, respectively, and bootstrap was −0.05 and 0.05. We see, however, that compared to a parameter such as Lab_ma, the absolute value of the real correlation coefficient only slightly exceeds the maximum value in randomized samples. This is also consistent with the fact that the ANOVA demonstrated no significant relationship between the harvesting year and this trait (*p* < 0.21, Supplementary File S4).

It should be noted that the highest negative values of the correlation coefficients (less than or equal to −0.6) are observed for the traits associated with the component a* of the L*a*b* space, which characterizes the grain redness. These are the traits Lab_ma, Lab_dCa_1, Lab_dCa_2 (mean value and values for two dominant colors). The greater the year, the lower the value of the component a*. This means that grain coat from early harvesting years have a redder tint compared to later harvesting years. Correlation coefficients smaller in absolute value are observed for the Cr component of the YCrCb scale, which is also related to the red component of the color: the larger the year, the smaller the value of the Cr component. Also of note, negative correlation between harvesting year and value/luminance/lightness components of different spaces HSV_mV (for YearRank *r* = −0.24), YCrCb_mY (for YearRank *r* = −0.15, Lab_mL (for YearRank *r* = −0.15). This indicates that the earlier the harvesting year, the lighter the color of the grain coat. In total, we observe 35 traits (all related to color characteristics), for which the relationship with the harvesting year is negative.

Significant positive correlation coefficients between the year of harvest and grain traits were found for ten traits, only one of them was the rugosity shape trait (as noted above). The rest were color traits. The highest in absolute value positive correlation coefficient (0.49) is observed for the HSV_mH trait, which is the mean value of the hue component of the HSV space. The H value is smaller for the earlier harvesting years and larger for the later ones.

For ten traits, the correlation coefficient with the harvesting year is close to 0 and insignificant according to randomization tests. Of these, six traits are size/shape characteristics and four are color characteristics (different components of dominant colors).

In the Supplementary File S5 we show significance of the ANOVA test and Pearson correlation for the relationship between seed trait and the harvesting year simultaneously. In general, traits with insignificant ANOVA test demonstrate lower absolute values of the correlation coefficients with the YearRank. At the same time, there are traits for which ANOVA demonstrates a significant relationship with the year of harvest, while the correlation coefficients are low (insignificant). These include all the size/shape traits excluding rugosity. These results demonstrate that the relationship between harvesting year and these traits do exist, but the significant trend is absent. Overall, the results of the ANOVA and the correlation analysis performed are in good agreement, and for the traits for which both the results of the ANOVA and the correlation analysis are significant, we can assume the presence of a trend associated with the year of harvest.

**Figure 3:**
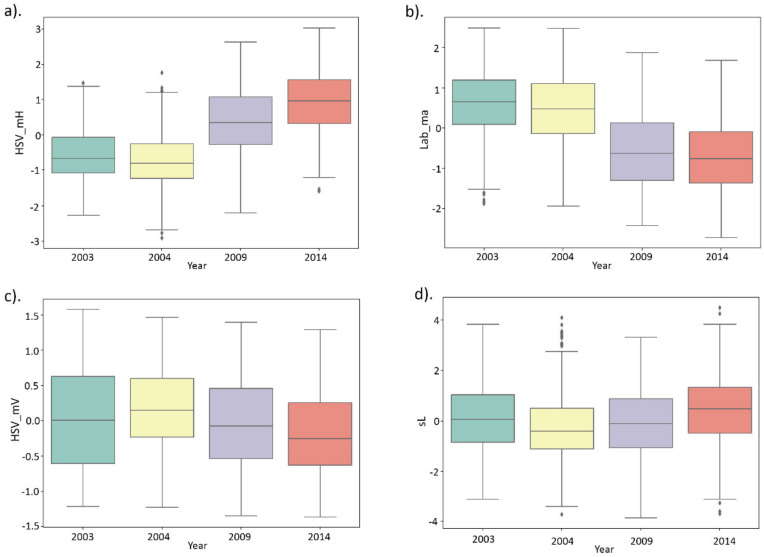
Bar plots for the distribution of the normalized HSV_mH (**a**), Lab_ma (**b**), HSV_mV (**c**) and sL (**d**) trait values (*Y* axis) in the seed samples from different harvesting years (*X* axis).

Figure 3 shows bar diagrams of the distributions of Lab_ma, HSV_mV (significant negative correlation with the year of harvest), HSV_mH (significant positive correlation with the year of harvest) and sL (no significant correlation with the year of harvest) for all genotypes depending on the year of harvest. Figure S4 of the Supplementary File S3 shows similar distributions for individual accessions for the same traits.

Both Figure 3 and Figure S4 show that for the traits Lab_ma, HSV_mV, the earlier harvest years correspond to higher trait values, and for the trait HSV_mH, on the contrary, lower values. For most genotypes, the differences between trait values in 2003 and 2004 are small, and for some genotypes the same is true for 2009 and 2014 (e.g., RILs ITM_001, ITMI_006, ITMI_007, ITMI_073, Figure S4). The greatest differences in traits are observed for the 2004 and 2009 harvests. In general, the trend of systematic decrease (traits Lab_ma, HSV_mV) or increase (trait HSV_mH) is clear in these figures.

Seed length (sL) demonstrate noticeable differences for different years, but no systematic year trend is observed. This is in agreement with the significant results for ANOVA test and the absence of the Pearson correlation between it and the harvesting year.

### 2.5. Analysis of Correlations between Germination and Grain Traits

**Figure 4:**
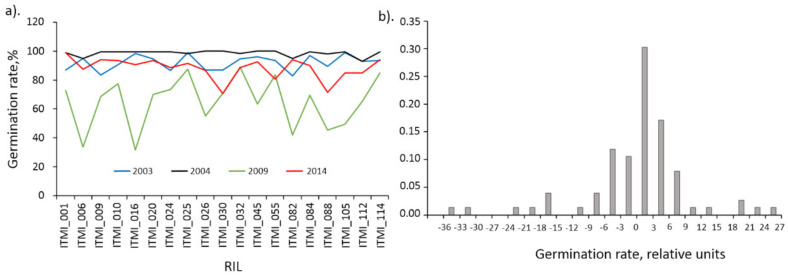
Germination of plants in the ITMI population for 19 genotypes evaluated in 2003, 2004, 2009, and 2014. (**a**) Germination values (*Y*-axis) for genotypes (*X*-axis) in different years shown by lines of different colors (see legend at bottom of graph); (**b**) Distribution of germination values after subtracting the annual average.

In order to investigate the relationships between seed germination and the other traits, we firstly evaluated the dependence of the germination on two factors: genotype and harvesting year using the ANOVA (Supplementary File S3, Tables S1 and S2). It turned out that for genotype this relationship was not significant (*p* < 0.98), while for the year of harvest it was significant (*p* < 1.2 ∙ 10^−15^). Indeed, for 2003 and 2004 the average germination values were 92.0% and 98.6%, for 2009 and 2014 64.9% and 88.2%, respectively (see Figure 4a). Data on germination of different accessions for 2009 are exceptional: they are systematically lower than the others and have a significantly greater variance. To eliminate the influence of the harvesting year on the germination values we subtracted the mean for the specific year from germination values.

The histogram of the distribution of germination values after the transformation is shown in Figure 4b. This distribution is broken down into a central part (from −15 to 15) and distant peaks (beyond this interval). Interestingly, the outlier peaks correspond mainly to 2009 data (ITMI_006, ITMI_016, ITMI_082, ITMI_088, ITMI_105 less than −15 and ITMI_032, ITMI_055, ITMI_114 greater than 15). Two values less than −15 are also observed for 2014 (ITMI_030 and ITMI_088). Since outliers can affect correlation coefficient estimates, we decided to exclude data for these accessions and years and consider only germination data that correspond to the central part of the distribution.

The relationship between germination rate and grain trait values was evaluated using Pearson correlation coefficient. Two randomization tests were performed to assess its significance, as described for the harvesting year: bootstrap and permutation. The results for all traits are given in Supplementary File S6.

A significant positive relationship was found only between germination values and hue mean values (HSV scale). Negative significant correlation coefficients were found for a number of traits that are associated with the grain redness. They can be divided into two groups. For the first group, the values of the correlation coefficients differ slightly from the threshold values. These are the features associated with value (intensity) on the HSV space (HSV_mV and HSV_dCV_1) and the red component of the RGB space (RGB_dCR_1, RGB_dCR_2). The second group (Table 2) includes the traits whose correlation coefficients with germination are more than 2 times different from the threshold. These are the traits associated with the red color component in YCrCb and L*a*b* spaces: YCrCb_dCCr_1, YCrCb_dCCr_2, YCrCb_mCr, Lab_dCa_1, Lab_dCa_2, Lab_ma.

For example, Table 2 shows, that HSV_mH has positive correlation coefficient (0.164) with germination values which is almost as twice as large as the maximal values obtained in permutation (0.080) and bootstrap (0.089) tests. This table also demonstrate that values of the Pearson correlation coefficients between Lab_ma and germination (−0.235) is negative and more than a twice the minimal values obtained in permutation (−0.100) and bootstrap (−0.086) tests. The same holds true for other traits listed in Table 2.

The relationship between the germination rate and the Lab_ma parameter is the most pronounced and negative. This means that the coat of seeds with lower values of germination rate have larger red component of their color. This is also true for the other parameter describing seed redness, Cr component of the YCrCb scale.

**Figure 5:**
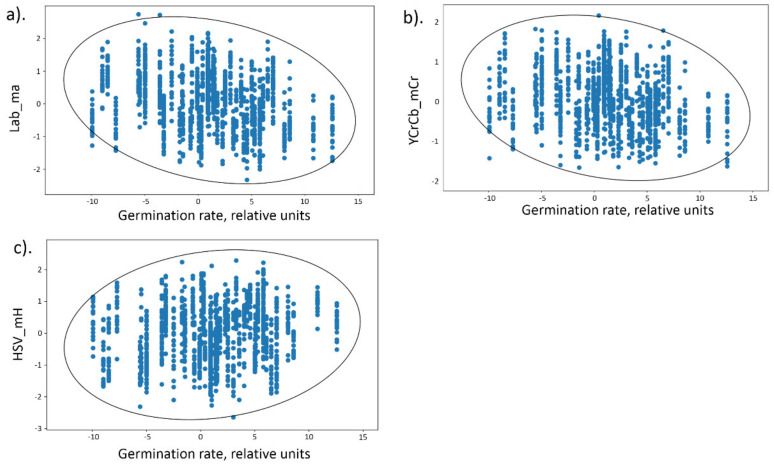
Scatter diagrams for germination values (*X* axis) and grain color traits (*Y* axis) of the RIL seeds (dots correspond to seeds): (**a**) Lab_ma, (**b**) YCrCb_mCr, (**c**) HSV_mH. Ellipsoids are shown at the 95% significance level.

Scatter diagrams for the germination values and three seed traits, Lab_ma, YCrCb_mCr and HSV_mH are shown in Figure 5. It can be seen that Lab_ma, YCrCb_mCr values demonstrate negative relationship with the germination; HSV_mH demonstrate positive relationship with the germination.

Dots on these diagrams correspond to seeds from the RILs and harvesting years for which germination values were estimated. From the Figure 5a,b it is apparent, the higher germination, the lower redness of grains, estimated as Lab_ma and YCrCb_mCr parameters (negative linear relationships between these traits and germination, see Table 2). Figure 5c demonstrates the positive relationship between HSV_mH parameter and germination: the higher the germination values, the greater the parameter value (see Table 2).

Note that no significant relationship with germination values was found for any of the grain shape/size traits (Supplementary File S6). The highest absolute value of the correlation coefficient is observed for the seed area sA (0.067), but it turns out to be insignificant.

## 3. Discussion

In our work, we analyzed the size/shape/color characteristics of wheat grains from ITMI population that were harvested in different years. To the best of our knowledge, such an analysis applied for seeds stored in genebank for several years, was performed for the first time. The statistical relationship of these characteristics with harvesting year and germination rate was investigated. The genetic component of variance was found to contribute significantly to the all traits studied, the harvesting year factor was significant for 80% of the traits, most of them are color traits. Most of the color characteristics demonstrated also a significant trend (linear correlation) between the trait and the harvesting year. We did not find significant linear correlations for the size/shape traits and the year of harvest.

It should be noted that the harvesting year factor is complex and can include both the influence of the environmental conditions on the grain properties depending on the year of cultivation, and the effect of the grain storage. The environmental component can be considered as a random factor (environmental conditions change randomly from year to year). The seed storage component, providing the identity of grain storage conditions, can be considered deterministic: grain storage time is constantly increasing. Thus, the seed storage result in a systematic change of traits, reduction or increase with the storage duration.

For the grain size/shape we observe significant ANOVA results but no correlations with the harvesting year (Figure 3d). Thus, grain size/shape properties affected by plant growing conditions in the greater extent than by the storage duration. This is in good agreement with the experimental observations about the influence of growing conditions on yield characteristics [24,25,26,27].

For most of the color characteristics, however, we observed both significant results for the ANOVA test and correlation analysis with the year of harvest. Consequently, these results show the dependence of grain coat color primarily on the storage duration: the longer the storage time, the lighter and more reddish grains become.

The color of the grains can be altered by various factors. Exposure to high temperatures and drying under microwave conditions [28,29,30] or roasting [31] results in darkening of grains and increasing of the red color component of their coats. This effect is known as browning [32] and is associated with seed injury [33]. The browning effect has been observed under conditions of controlled seed deterioration in lentil [22] and cucumber [20]. In the last two studies, the color characteristics were evaluated based on the RGB index, the average value of the intensities of the R,G,B components of the grain color. It was shown that under controlled deterioration process, the RGB index decreased, which reflected the darkening of the grain coat.

As in the above-mentioned works, we showed that in wheat the redness of the grain coat increased with increasing storage duration. However, in contrast to browning, we observed a significant lightening effect, although rather weak: with increasing storage time the color components related to the luminance/lightness of the grain coat increased. A possible reason for this is the storage conditions at negative temperatures (−18 °C), as opposed to artificial aging by extreme temperature/humidity exposures [23]. This suggests that artificial aging cannot fully model biochemical processes during seed deterioration at genebank conditions. Interestingly, the differences for two types of seed deterioration resulted in different changes in seed molecular components [34].

Our data are in good agreement with the results of evaluation of the properties of einkorn wheat grains stored under mild conditions at different temperatures for 360 days [35]. Authors show that grain color characteristics, including lightness and redness, are positively correlated with storage time, which is in agreement with our results. We observed no significant linear correlations between grain shape/size and storage time, while Kibar [36] showed a negative correlation between wheat and corn grain size and storage time up to 90 days: grain size decreased with increasing storage time. It should be noted, however, that in the experiment by Kibar [36] grains were harvested at the same season and belonged to the same genotype, while in our experiment we studied grains of different harvesting years. Our results can be explained by the fact that the influence of growing conditions makes a more significant contribution to the characteristics of grain size than the duration of grain storage.

Our data also showed that the intensity of the red color component of the grain coat has a negative relationship with the germination rate of seeds. A number of experiments evaluating the effect of seed color on the germination rate show similar results. The work of Janampa-Santome et al. [37] shows that dark-colored seeds of the high-Andean crop *Lepidium meyenii* (Maca) have lower germination compared to the lighter ones. A statistically significant effect of negative relationship between color intensity of seeds without coat and germination percentage was observed in accelerated aging experiment for *M. sativa* and *O. viciifolia* [21].

These dependencies can be explained by the relationship between the physiological properties of seeds and the biochemical composition of their coat. Seed color is defined by the presence of various pigments in its coat [38]. Among others are lignans [39], phenolic acids [40], carotinoids, including lutein and their derivatives [41]. These metabolites determines seed germination [42,43]. Delayed germination has been related to the presence of condensed and oxidized tannins (brown pigment), which may harden seed coat. Seed color could also be associated with variation in other secondary metabolites, which may also affect its germination ability [44]. Note that similar molecular mechanisms play an important role in the resistance of grains to preharvest sprouting: it is well known that red grains are more resistant to it than white ones [45,46]. During storage, various enzymes could affect these metabolites. For example, lutein is prone to degradation by a co-oxidation process that involves the enzyme lipoxygenase in food products [47] which results in loss of color. It is possible that similar processes of yellow pigment degradation due to lipoxygenase activity could be involved in decreasing of their concentration in seed coat and, consequently decreasing in the yellow color component and increasing the red one in our seed samples.

Our results demonstrate that imaging and digital image processing make it possible to estimate with a high degree of detail the characteristics of both grain size/shape and color. This shows that such techniques are highly accurate and can be used not only to evaluate the properties of grains but also to predict their physiological state [20,30]. The great advantage of such methods for monitoring the physiological condition of grains is their noninvasiveness and affordability for genebanks. Our results, however, show a high variability in grain traits within and between RILs (see Supplementary File S3, Figures S1, S2 and S5). This can strongly affect the accuracy of the prediction of the physiological status of the grain (e.g., germination). In the present work, we have only demonstrated significant general trend in grain properties as a function of storage time. Accurate determination of grain properties may require the development of better measurement methods (e.g., multispectral [48]) and advanced mathematical methods [30].

In our work, it was impossible to compare the characteristics of grains after several years of storage with those immediately after harvesting. No phenotyping of the grains was carried out at the time of harvesting. This does not allow a direct assessment of the change in the properties of the grains in relation to their original data. Such an analysis would be very informative, because some recombinant lines of bread wheat (*Triticum aestivum* L.) have seed coats colored differently. This problem could be solved, however, by introducing the widespread use of digital phenotyping technologies for plant samples before they are placed in storage or genebanks [49].

## 4. Materials and Methods

### 4.1. Plant Material

We analyzed seeds from the 44 recombinant inbred lines from the mapping population of bread wheat (*Triticum aestivum* L.) of the International Triticeae Mapping Initiative (ITMI). List of RILs provided in the Supplementary File S1 (‘RILs’ tab). The ITMI mapping population was obtained by crossing *T. aestivum* (var. Opata 85) with the synthetic hexaploid spring wheat W7984. For details see Arif et al. [50]. Plants of each RIL were grown in the 2003, 2004, 2009 and 2014 year seasons at the experimental fields of the Leibniz Institute of Plant Genetics and Crop Plant Research (IPK) in Gatersleben, Germany (latitude 51°49′19.74″ N, longitude 11°17′11.80″ E, 110.5 m.a.s.l., black soil of clayey loamy type). After harvest the seeds were stored at IPK genebank at −18 ± 2 °C and 8 ± 2% seed moisture content.

### 4.2. Germination Evaluation

Percentage of the seed germination was determined for 19 RILs (ITMI_001, ITMI_006, ITMI_009, ITMI_010, ITMI_016, ITMI_020, ITMI_024, ITMI_025, ITMI_026, ITMI_030, ITMI_032, ITMI_045, ITMI_055, ITMI_082, ITMI_084, ITMI_088, ITMI_105, ITMI_112, ITMI_114) originated from seasons 2003, 2004, 2009, and 2014. Tests were performed for each entry in four replications (independent experiments) of 50 seeds per line. The seeds were placed in pleated filter paper strips (Munktell-Filtrak GmbH, Germany; 50 pleats, 2000 mm × 110 mm/20 mm depth). Pleated paper strips were inserted in plastic boxes with a flat filter paper (Munktell-Filtrak GmbH, Germany; C 160, 105 mm × 155 mm) on the ground for evenly water distribution below pleated paper strips. The boxes were covered with clear plastic lids against evaporation. The water saturation was 85% of the maximum capacity from the pleated paper strip and the flat ground paper. Germination tests were performed in a climate chamber (Kühlanlagenbau Dresden GmbH, Germany). The temperature was constant at 20 ± 2 °C and the phase of illumination was 8 h per day. On day 8 the number of seedlings showing a normal appearance (normal germination) according to ISTA [51] were recorded.

### 4.3. Imaging

**Figure 6:**
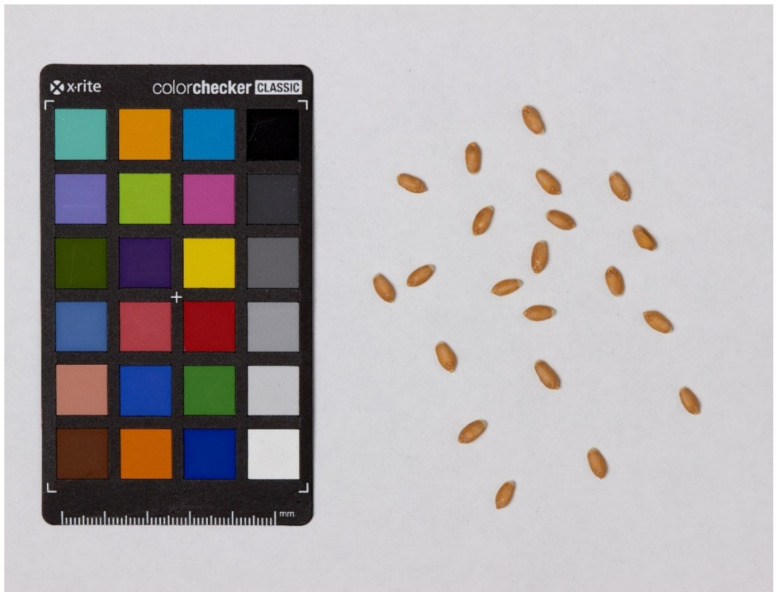
Image of grains of the ITMI_110 RIL.

We obtained one seed image for each RIL and harvesting year. The number of grains per image varies from 17 to 20. Wheat grains were scattered without touching on a white background (A4 sheet of paper). Each image contains a ColorChecker Mini Classic target (https://calibrite.com/us/product/colorchecker-classic-mini/; accessed on 10 November 2021) for color correction. This correction allows for avoiding color shifts in the images, which result from differences in the lighting conditions. The lighting was adjusted to avoid shadows. RGB images of the seeds were taken with a digital camera Canon EOS 600D, with Canon EF 100 mm f/2.8 Macro USM lens and saved in JPG or PNG format. An example of the resulting image is shown in the Figure 6.

### 4.4. Image Processing

Image processing was performed using the SeedCounter desktop version [52], supplemented with a module for calculating color characteristics described below. The application is implemented using the OpenCV [53] library in the Java programming language.

The main steps of image processing include:

1. ColorChecker recognition, scaling and color correction;
2. Image color segmentation;
3. Identification of contours;
4. Filtration of obtained contours, grain recognition;
5. Evaluation of grain size and shape characteristics;
6. Evaluation of color characteristics of grains.

The first stage was implemented based on the algorithm we described earlier [54]. Stages 2–4 of the analysis implemented as in the paper of Komyshev et al. [52]. The details of steps 5 and 6 will be described below.

### 4.5. Evaluation of Grain Size and Shape Characteristics

Seed size characteristics include length (sL), width (sW), and projected area in the image (sA). The method for estimating these parameters is described earlier [52]. Additionally, we evaluated seed circularity (sCi), roundness (sRo), rugosity (sRg), Solidity (sSo) [55,56]. Circularity and roundness reflect how close the shape of the grain contour is to the shape of the circle, but calculated differently.

Circularity is a measure of the similarity of a plane figure to a circle and calculated by the formula of Cervantes et al. [55]:

$$sCi=\frac{4\pi \times area}{perimeter^{2}},$$

where *area* and *perimeter* are figure’s area and perimeter, respectively. A circle is the figure with the smallest perimeter for a given area, a figure other than a circle will have a circle index less than unity.

For seed contours with many small convexities, the perimeter increases and the circularity index takes lower values. In these cases, it is useful to use the roundness index to describe the closeness of the shape to a circle, because this value does not depend on the roughness of the contour line. This index is calculated as the ratio of the area of the contour to the square of the length of the major axis of rotation. The *Major axis* for the circle is 2*R*, where *R* is its radius; therefore, roundness is calculated following Cervantes et al. [55]:

$$sRo=\frac{4\times area}{\pi \;{\left[Major\;axis\right]}^{2}}.$$

For a shape other than a circle, the denominator will increase and the index takes on values less than 1.

Rugosity (sRg) is defined as the ratio of the contour perimeter to the convex perimeter [55]:

$$Rg=\frac{Ps}{Pc},$$

where 𝑃𝑠 is the perimeter of the contour, and 𝑃𝑐 is the convex perimeter of the contour, also known as the convex hull, which is the smallest convex shape that contains all pixels of the contour.

Solidity (*sSo*) is the ratio of the area of the contour to the area of its convex hull [56]:

$$sSo=\frac{Contour\;Area}{Convex\;Hull\;Area}.$$

### 4.6. Evaluating the Color Characteristics of the Grains

To describe the color characteristics of the grains, we used four color spaces: RGB, HSV, L*a*b*, and YCrCb [16,57,58]. Each of them represents color as three components. The values of the components of one space can be obtained by transforming the values of the components of the other. RGB color space is widely known and represent color in the intensities of the red (R), green (G) and blue (B) components. The HSV space represent color as combination of the hue (H, the similarity to red, yellow, green, and blue colors depending on the value), saturation (S, the higher saturation, the more pure is the color), and value/intensity (V, the higher the value, the brighter the color) components. CIE L*a*b* space expresses color as combination of the lightness (L*, the greater value, the lighter is the color) and color components a* (smaller values correspond to green, larger to red tones of the color) and b* (smaller values correspond to blue, larger values correspond to yellow tones). YCrCb space represent color as a combination of luminance or luma (Y, the greater value, the higher luminance), difference between the red component and luma (Cr, the greater value, the close color to red) and difference between the blue component and luma (Cb, the greater value, the close color to blue).

The advantage of using color representation by HSV, L*a*b*, and YCrCb spaces is their ability to decompose the color into independent brightness/lightness/luminosity (V, L*, Y) and color components. This allows to describe seed coat coloration more robust, despite the differences in the illumination of its parts due to complex 3D shape.

In our work the color components values vary from 0 to 255, except the H component of the HSV space, whose values range was from 0 to 180.

The first type of descriptors are the average intensity values of the components for the grain pixels. These characteristics were calculated in two steps. First, the mean values and standard deviations for the components were estimated for all pixels of the grain images. After that, pixels with component intensities deviating from the mean by more than 3 standard deviations were discarded. For the remaining pixels, the mean component values were again calculated and used. In the following, the descriptors of the average component values will be designated by the small letter m. For example, for the RGB color space these are three parameters RGB_mR (mean for the intensity of the red component, R), RGB_mG (mean for the intensity of the green component, G), RGB_mB (mean for the intensity of the blue component, B); for other spaces the designations are similar.

The next set of features are the dominant grain colors. The dominant color descriptors provide a determination of representative colors in an image or its region [59]. To compute these descriptors, all pixels related to the grain image in the color space are clustered by component intensity using the *k*-means method where *k* is the number of representative clusters. In the present work, *k* = 3. The resulting clusters *i* = 1, …, *k* are ranked by the fraction of pixels that belong to them. For each cluster, the centroid represents the dominant color. For each such centroid, the intensities of color components were determined (for example, for RGB space, they are RGB_dCj_i, where j is a color component, and i is the number of the dominant cluster, for example, RGB_dCR_1 is the red component, R, of the first dominant color in RGB space). The use of three dominant colors allows a more precise estimation of grain color tones.

As a result, 3 characteristics of size, 4 characteristics of shape and 48 characteristics of color were determined for each grain. Full list of characteristics with descriptions provided in the Supplementary File S1 (‘Traits’ tab). These characteristics were calculated for each seed from 44 RILs and each of the 4 harvesting years; 3681 seeds were analyzed in total.

### 4.7. Cluster Analysis and LDA

Cluster analysis was performed using unweighted pair group method with arithmetic mean (UPGMA) and the Euclidean distance between seed traits. Linear discrimination analysis (LDA or canonical variates analysis) was performed separately for grain size/shape and color traits. We used the Python Scipy library (https://scipy.org/; accessed on 5 May 2021) for clustering (scipy.cluster.hierarchy and scipy.spatial.distance modules) and PAST v.3 [60] for LDA. The ranges of the seed traits varied significantly (for example for seed length/width and area); therefore, they were standardized before cluster analysis and LDA.

### 4.8. Analysis of Variance and Correlation Analysis

To test the hypothesis about the relationship between the harvesting year (i.e., the duration of seed storage) and the characteristics of grain size/shape and color, we used several statistical approaches, described below.

Analysis of variance (ANOVA) was performed using harvesting year and genotype as independent variables and the seed traits as dependent ones. We used Pearson’s correlation coefficient to identify linear relationship (trends) between the crop year and the value of the trait. However, since the harvest years (2003, 2004, 2009, and 2014) are separated by unequal time intervals, we applied three coding options for this variable. The first coding option (Year01) assigned a value of 0 for years 2003 and 2004 and a value of 1 for years 2009 and 2014. This coding allows us to divide crop years into earlier (2003, 2004) and later years (2009, 2014). The second coding option (YearRank) assigned a rank to different years in ascending order (respectively, 1 for 2003, 2 for 2004, 3 for 2009, and 4 for 2014). This method equalizes the difference in time intervals between harvest years. The third coding option (Year) used the value of the harvesting year as input for correlation analysis. Since the analysis of variance showed that the contribution of the genetic component to the variance of grain characteristics is significant, in order to eliminate this effect, we subtracted the mean of the corresponding genotype from the values of the variables before the correlation analysis. To assess the significance of the correlation coefficient estimates, we used bootstrap analysis and permutation test (2000 replicates in each case), and calculated the Pearson correlation coefficient with the YearRank parameter. The maximum value of the correlation coefficient and the minimum value were determined for all random samples. The relationship between a trait and YearRank was considered significant at *p* < 2∙10^−3^ if the real correlation coefficient was either less than the minimum or greater than the maximum value in both tests. The ANOVA and correlation analysis were performed using the JACOBI4 package [61,62].

Evaluation of the statistical relationship between seed germination of different genotypes and their characteristics was carried out on the basis of linear Pearson correlation coefficients. We preliminarily evaluated the contribution of harvesting year and genotype to the variance of germination values. Since the crop year had a significant contribution to germination variance, we subtracted the average for the corresponding year from the germination values for each genotype and year. Some outlier values were detected after that in the germination data. After removing the RIL and year data for these outlier values, the number of analyzed seeds used for the correlation analysis became 1363. Correlation analysis between grain traits and germination percentage was performed in the same way as was done for the harvesting year.

## 5. Conclusions

Analysis of grain size, shape and color of 44 RILs from ITMI population of different harvesting years allowed us to characterize seed traits for these accessions. It was shown that genotype significantly affects the variability of all grain traits, and the harvesting year affects about three-fourth of them (including all traits of shape and size). Evaluation of the linear correlation between grain size/shape traits showed no such correlation, which together with the ANOVA results suggests that these traits are significantly influenced by plant growing conditions rather than the duration of seed storage.

For the majority of color traits, significant correlations with the year of harvest were observed, which demonstrates the trend in their change associated with the duration of storage. For the majority of color traits these relationships are negative. The most pronounced negative relationship is observed for the traits associated with the redness of the seed coat: the earlier the year of harvest, the greater is the red component of the color. At the same time, the lightness of grain coat increases with increasing storage duration: the earlier the harvesting year, the lighter is the grain coat.

Analysis of linear correlations between germination of wheat seeds of different ITMI RILs and the seed characteristics revealed a negative linear relationship between the red component of coat color and germination: the larger the red color component, the lower their germination rate.

Our results demonstrate manifestations of metabolic changes in the coat of grains associated with prolonged storage and their relationship with the reduction of seed viability.

## Figures and Tables

**Table 1:** Traits of grain color with the most pronounced correlation with the harvesting year (YearRank coding) and estimates of the Pearson correlation coefficients for them in the real sample (*r*) and their minimum (*r*_min_) and maximum (*r*_max_) values for the permutation and bootstrap tests.

Trait	*r*, Real Data	Permutation, *r*_min_	Permutation, *r*_max_	Bootstrap, *r*_min_	Bootstrap, *r*_max_
HSV_mH	0.511	−0.051	0.052	−0.051	0.067
YCrCb_mCr	−0.509	−0.052	0.058	−0.048	0.058
YCrCb_dCCr_2	−0.513	−0.063	0.052	−0.052	0.059
Lab_dCa_2	−0.599	−0.063	0.051	−0.055	0.048
Lab_dCa_1	−0.610	−0.062	0.050	−0.057	0.066
Lab_ma	−0.639	−0.067	0.058	−0.056	0.056

**Table 2:** Grain color traits with the most pronounced correlation with germination values, estimates of the Pearson correlation coefficients for them in the real sample (*r*) and their minimum (*r*_min_) and maximum (*r*_max_) values for the permutation and bootstrap tests.

Trait	*r*, Real Data	Permutation, *r*_min_	Permutation, *r*_max_	Bootstrap, *r*_min_	Bootstrap, *r*_max_
HSV_mH	0.164	−0.088	0.080	−0.082	0.089
YCrCb_dCCr_1	−0.177	−0.077	0.100	−0.088	0.104
YCrCb_dCCr_2	−0.190	−0.082	0.096	−0.088	0.095
YCrCb_mCr	−0.197	−0.084	0.094	−0.077	0.104
Lab_dCa_1	−0.201	−0.100	0.124	−0.083	0.090
Lab_dCa_2	−0.224	−0.101	0.096	−0.088	0.091
Lab_ma	−0.235	−0.100	0.083	−0.086	0.114

## Supplementary Materials

The following are available online at www.mdpi.com/xxx/s1/. Supplementary files. ‘Supplementary File S1.xlsx’ (Excel format): Lists of RILs used in analysis (‘RILs’ tab); list of traits with their description (‘Traits’ tab); trait means for the entire dataset (‘TraitMeans’ tab). ‘Supplementary File S2.xlsx’ (Excel format): Pearson correlation coefficients between seed traits for entire dataset with *p*-values. ‘Supplementary File S3.docx’ (Word format): Supplementary Figures S1–S4; Supplementary Tables S1 and S2. ‘Supplementary File S4.xlsx’ (Excel format): ANOVA results for traits (dependent variables), genotype and year of harvesting (independent variables). ‘Supplementary File S5.xlsx’ (Excel format): Correlation analysis results between seed traits and harvesting year. ‘Supplementary File S6.xlsx’ (Excel format): Germination data for 19 RILs and correlation analysis results between seed traits and germination rate.

## Abbreviations

ANOVA	Analysis of variance
CIE	International Commission on Illumination
HSV	Color space described by Hue, Saturation and Value components
ISTA	International Seed Testing Association
ITMI	International Triticeae Mapping Initiative
L*a*b*	Color space described by Lightness, a* and b* components
LDA	Linear discrimination analysis
RGB	Color space described by Red, Green and Blue components
RIL	Recombinant inbred line
UPGMA	Unweighted pair group method with arithmetic mean
YCrCb	Color space described by luminosity, Cr and Cb components
