# Supplementary material for: Relationship between the Characteristics of Bread Wheat Grains, Storage Time and Germination
Source: Plants (Basel). 2021 Dec 23;11(1):35. doi: 10.3390/plants11010035 (PMC8747681; doi:10.3390/plants11010035)
Supplement: Supplementary file 1 [file plants-11-00035-s001.zip › Supplementary File S3.pdf]

Supplementary file S3

for the paper “Relationship between the characteristics of bread wheat grains, storage time and germination“ Afonnikov D.A., Komyshev E.G., Efimov V.M., Genaev M.A., Koval V.S., Gierke P.U., Börner A.

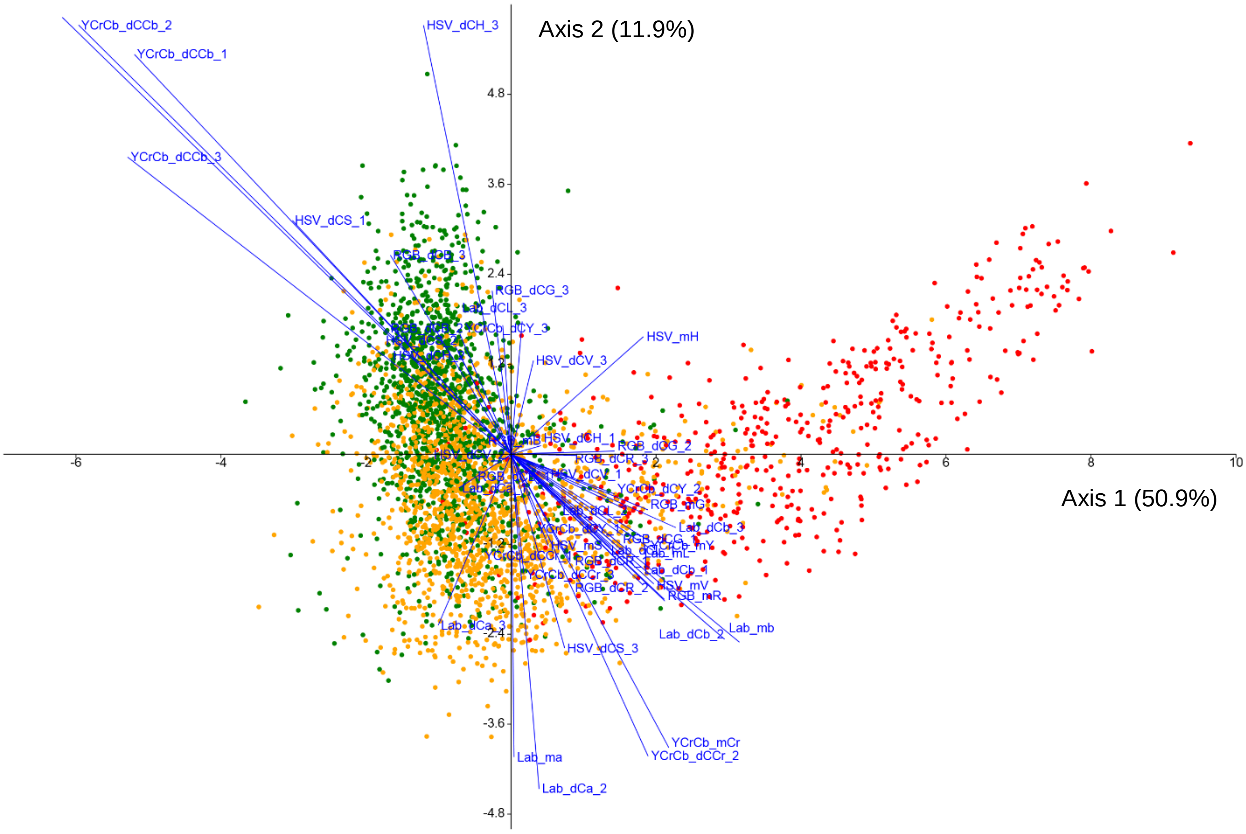

Figure S1. LDA biplot for seeds from 44 RILs in the plane of two first main axes of the color traits space. The fractions of variance for two axes are shown in parentheses.

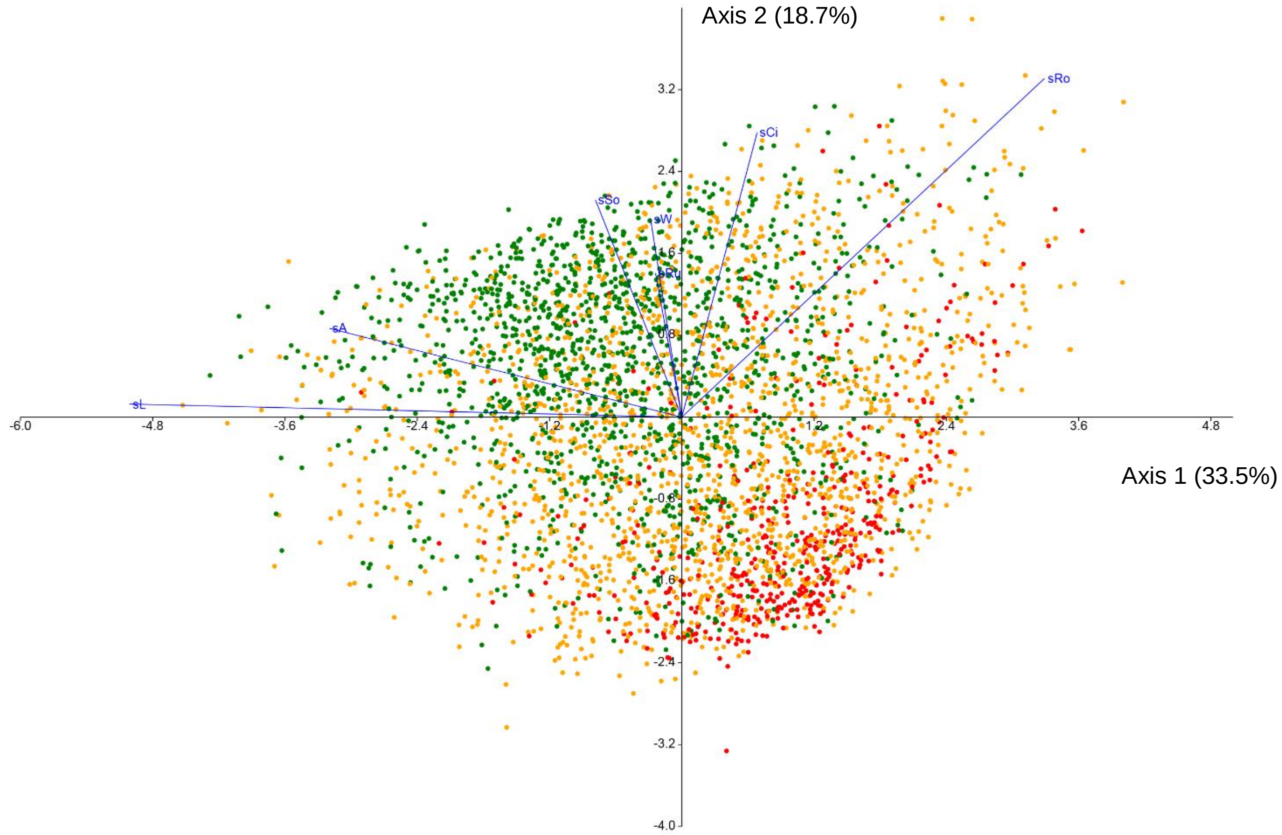

Figure S2. LDA biplot for seeds from 44 RILs in the plane of two first main axes of the size/shape traits space. The fractions of variance for two axes are shown in parentheses.

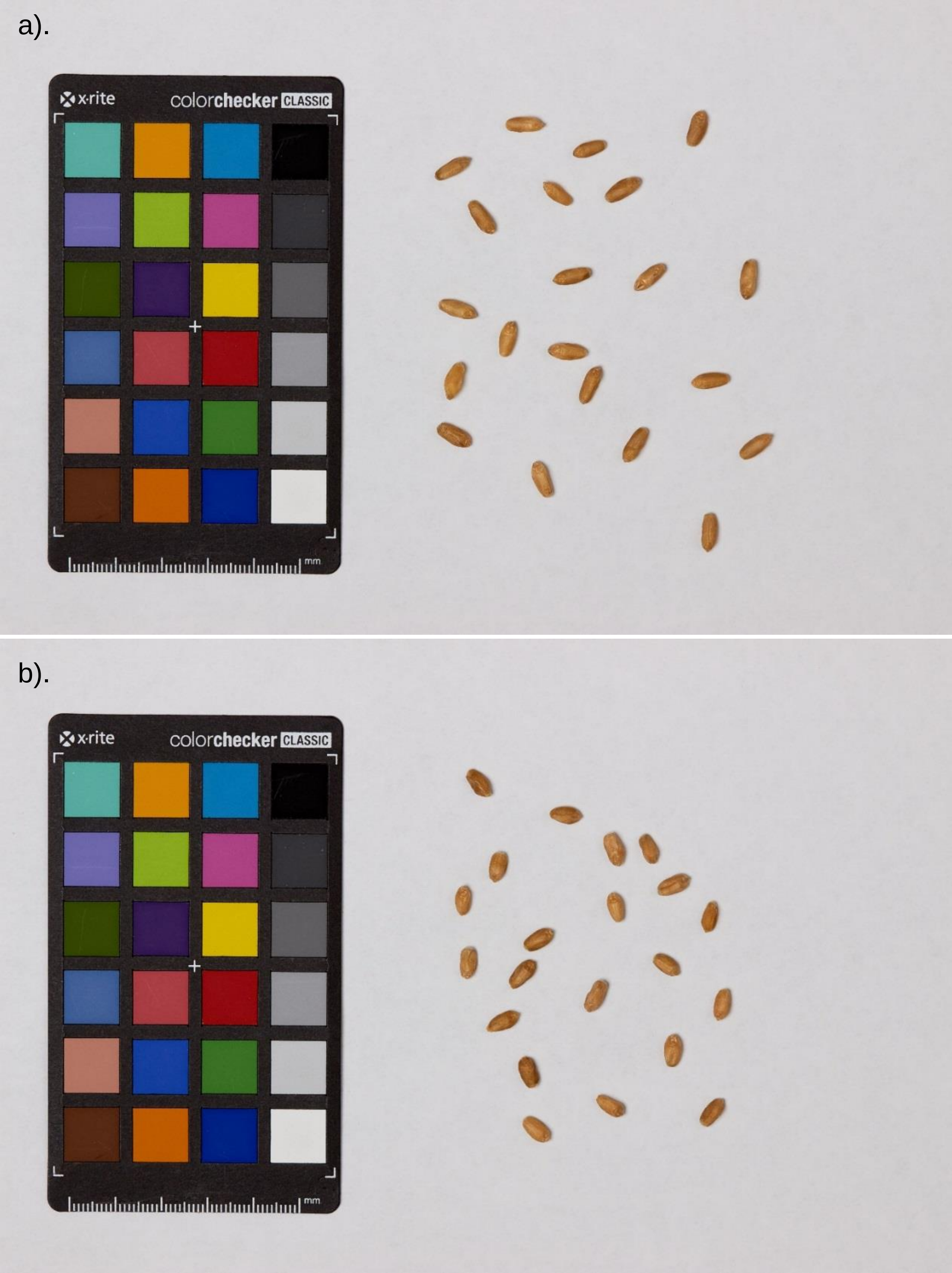

Figure S3. Images of grains from two RILs: ITMI_016 (a) and ITMI_028 (b).

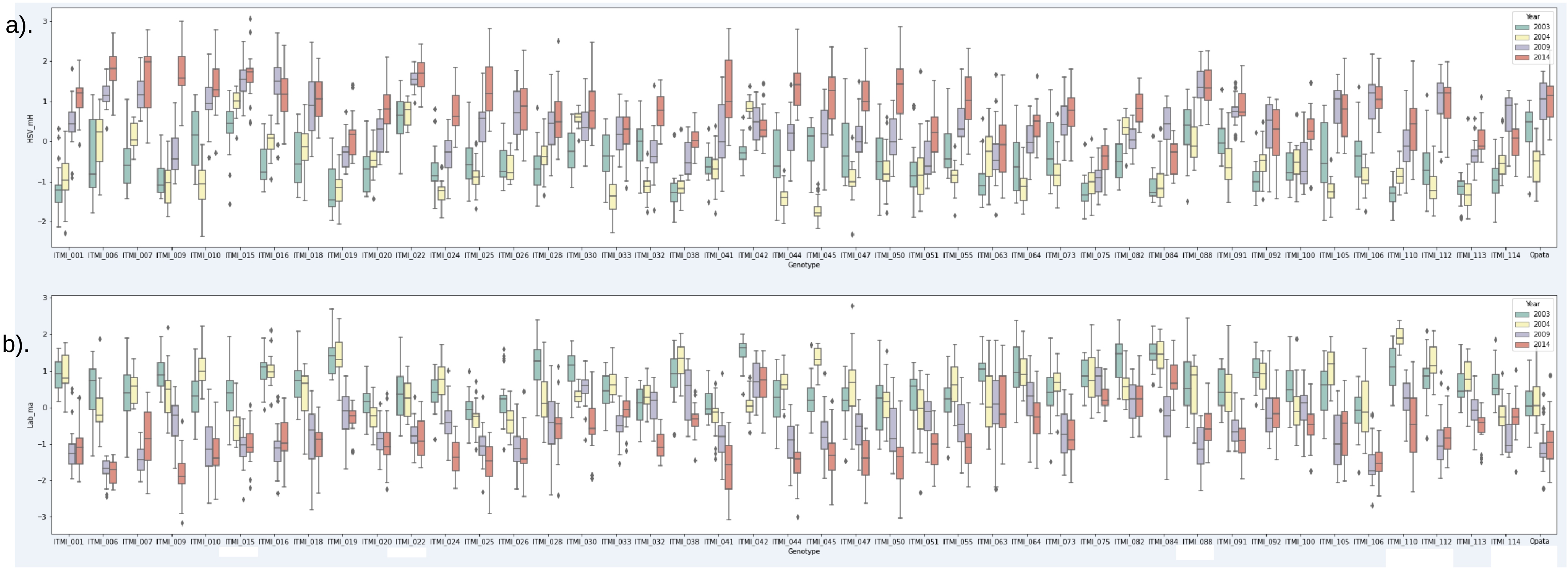
a).
HSV_mH
Year
2003
2004
2009
2014
ITMI_001 ITMI_006 ITMI_007 ITMI_009 ITMI_010 ITMI_015 ITMI_016 ITMI_018 ITMI_019 ITMI_020 ITMI_022 ITMI_024 ITMI_025 ITMI_026 ITMI_028 ITMI_030 ITMI_033 ITMI_032 ITMI_038 ITMI_041 ITMI_042 ITMI_044 ITMI_045 ITMI_047 ITMI_050 ITMI_051 ITMI_055 ITMI_063 ITMI_064 ITMI_073 ITMI_075 ITMI_082 ITMI_084 ITMI_088 ITMI_091 ITMI_092 ITMI_100 ITMI_105 ITMI_106 ITMI_110 ITMI_112 ITMI_113 ITMI_114 Opata
Genotype
b).
Lab_ma
Year
2003
2004
2009
2014
Genotype

c).

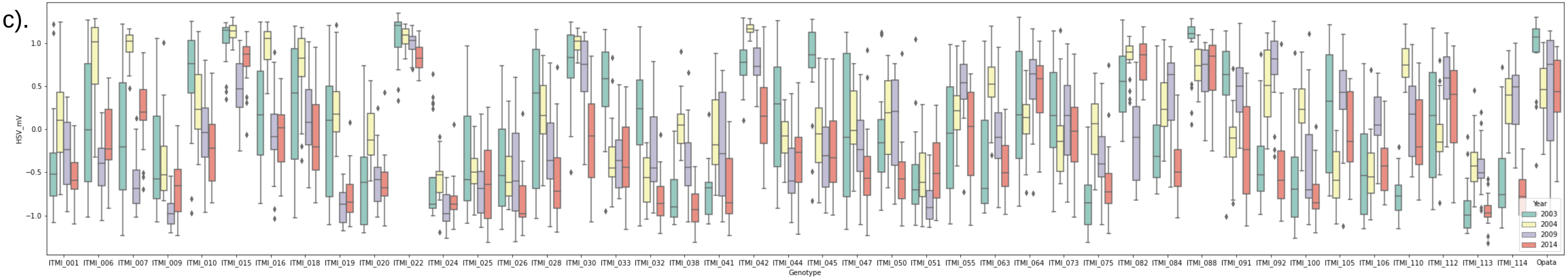

d).

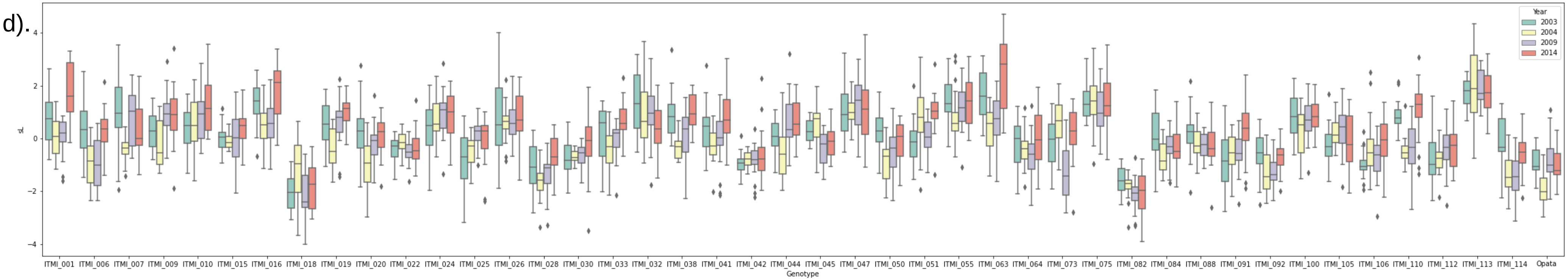

Figure S4. Bar plots for the distribution of the HSV_mH (a), Lab_ma (b), HSV_mV (c) and sL (d) trait values (Y axis) in the seed samples from different harvesting years and accessions (X axis; green bars for 2003, yellow bars for 2004, purple bars for 2009, red bars for 2014).

Table S1. The ANOVA results for the ITMI seed germination and the year of harvest.

Variance component	Sum of Squares	df	Mean square	F	P-value	F threshold
Between groups	12218.868	3	4072.956	41.238	$1.3 \cdot 10^{-15}$	2.732
Within groups	7111.158	72	98.766			
Total	19330.026	75				

Table S2. The ANOVA results for the ITMI seed germination and the genotype.

Variance component	Sum of Squares	df	Mean square	F	P-value	F threshold
Between groups	2101.776	18	116.765	0.3863	0.9858	1.788
Within groups	17228.250	57	302.250			
Total	19330.026	75				
